# Postdischarge Alcohol Cessation and Psychiatric Referrals in Alcoholic Liver Disease

**DOI:** 10.1001/jamanetworkopen.2025.11619

**Published:** 2025-05-20

**Authors:** Shu-Yen Chan, Yee Hui Yeo, Hyunseok Kim, Natchaya Polpichai, Yueh-Ting Tsai, Peng-Sheng Ting

**Affiliations:** 1 Department of Internal Medicine, Weiss Memorial Hospital, Chicago, Illinois; 2 Department of Medicine, Cedars-Sinai Medical Center, Los Angeles, California; 3 Karsh Division of Gastroenterology and Hepatology, Cedars-Sinai Medical Center, Los Angeles, California; 4 Department of Emergency Medicine, Chung Shan Medical University Hospital, Taichung, Taiwan; 5 Division of Gastroenterology and Hepatology, Tulane University School of Medicine, New Orleans, Louisiana; 6 University Medical Center of New Orleans, New Orleans, Louisiana

## Abstract

This cohort study evaluates nationwide trends of postdischarge alcohol relapse prevention treatment for hospitalized individuals.

## Introduction

Alcohol-associated liver disease (ALD) is a leading cause of liver-related mortality globally^1^ and is associated with high rates of recidivism. Rogal et al^2^ demonstrated a significant reduction in hepatic decompensation and all-cause mortality among those who received alcohol use disorder (AUD) treatment. However, there is a lack of data on the temporal trends, prevalence of treatment utilization, and disparities across sociodemographic subpopulations in the US. We aim to evaluate nationwide trends of postdischarge alcohol relapse prevention treatment for hospitalized individuals.

## Methods

This population-level cohort study used longitudinal electronic health records from the TriNetX dataset. It was exempt from review and informed consent per the Common Rule and followed the STROBE reporting guideline. We included individuals aged 21 years or older hospitalized with ALD, including alcohol-associated hepatitis or cirrhosis, from January 1, 2014, to December 31, 2023, identified through diagnosis codes (eTable in Supplement 1). The primary outcome was the incidence of common pharmacotherapy prescriptions for alcohol cessation (baclofen, naltrexone, acamprosate, disulfiram, gabapentin, and topiramate), psychiatric referrals, or outpatient psychiatric encounters within a year after discharge. Incidence trends over 10 years were analyzed using Jointpoint Regression Software. Subgroup analyses based on cirrhosis status and sex were performed. To detect significant shifts, we calculated the annual percentage change (APC) and average annual percentage change (AAPC) with 95% CIs. Advanced analyses of nonparallel AAPC comparisons between groups were conducted using permutation tests and parametric methods. For more details, see the eMethods in Supplement 1.

## Results

Among 117 million patients in the database, the number of individuals with ALD-related hospitalization tripled, from 11 539 in 2014 to 37 548 in 2023. Postdischarge pharmacological cessation prescriptions rates in individuals with ALD-related hospitalization increased from 12.6% in 2014 to 23.9% in 2023, with a greater increase between 2014 and 2016 (APC, 17.3%) than from 2016 to 2023 (APC, 5.1%). In contrast, psychiatric referrals or outpatient follow-ups started at a lower baseline 4.8% in 2014, initially declined (APC, −6.1% from 2014 to 2017), and then significantly increased from 2017 onward (APC, 8.7%; 95% CI, 5.1%-12.5%), peaking at 6.5% in 2023. Mortality rates among hospitalized individuals with ALD decreased from 16.8% in 2014 to 13.9% in 2017, increased to 15.6% in 2020, and then declined to 13.3% by 2023 (Figure).

**Figure.**
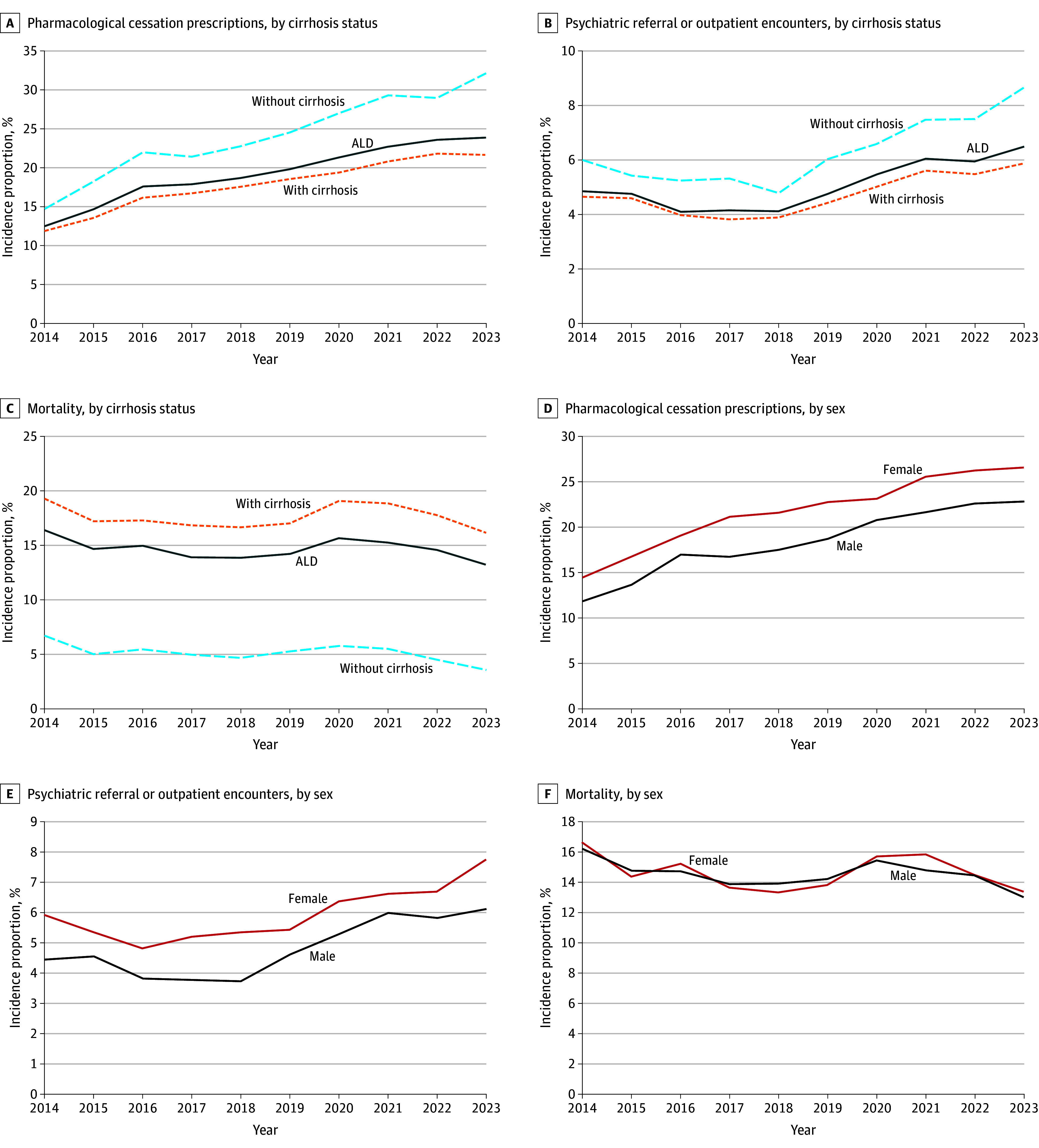
Trends in Postdischarge Pharmacological Cessation Prescriptions, Psychiatric Referrals or Outpatient Encounters, and Mortality Among Patients With Alcohol-Associated Liver Disease (ALD), 2014-2023, by Cirrhosis Status and Sex

Subgroup analysis revealed that individuals with alcohol-associated cirrhosis had lower rates of both pharmacological and psychiatric interventions and higher mortality vs those without cirrhosis. Pharmacological cessation prescriptions significantly increased in both cirrhosis and noncirrhosis groups over the past decade, but only patients without cirrhosis showed a significant increase in psychiatric follow-ups. Women exhibited higher rates of pharmacological cessation prescriptions and psychiatric follow-ups than men, although men exhibited steeper increases in both interventions. Although both sexes demonstrated significant increases in postdischarge pharmacological prescriptions, there were no significant changes in psychiatric follow-ups or mortality over the 10-year period (Table).

**Table.** Trends of Pharmacological Cessation Prescriptions, Psychiatric Referrals or Outpatient Encounters and Mortality in Patients With ALD Stratified by Cirrhosis Status and Sex With APC, AAPC, and Nonparallel Comparisons

Patient group	Pharmacological cessation prescriptions		Psychiatric referrals or outpatient encounters		Mortality	
Individuals with ALD						
Trend 1, years	2014 to 2016		2014 to 2017		2014 to 2017	
APC, % (95% CI)	17.3 (9.0 to 26.2)^a^		−6.1 (−15.2 to 3.9)		−5.1 (−16.1 to 7.5)	
Trend 2, years	2016 to 2023		2017 to 2023		2017 to 2021	
APC, % (95% CI)	5.1 (4.0 to 6.1)^a^		8.7 (5.1 to 12.5)^a^		3.1 (−8.9 to 16.7)	
Trend 3, years	NA		NA		2021 to 2023	
APC, % (95% CI)	NA		NA		−7.2 (−27.6 to 19.0)	
AAPC 2014-2023, % (95% CI)	7.7 (6.2 to 9.1)^a^		3.5 (0.4 to 6.8)^a^		−2.0 (−5.9 to 2.0)	
AAPC comparison (pharmacology vs psychiatric)	4.1 (0.6 to 7.7)^a^					
AAPC comparison (pharmacology vs mortality)	9.7 (5.4 to 13.9)^a^					
AAPC comparison (psychiatric vs mortality)	5.5 (0.4 to 10.6)^a^					
Individuals with ALD, by cirrhosis status	Cirrhosis	Noncirrhosis	Cirrhosis	Noncirrhosis	Cirrhosis	Noncirrhosis
Trend 1, years	2014 to 2016	2014 to 2016	2014 to 2017	2014 to 2018	2014 to 2018	2014 to 2023
APC, % (95% CI)	16.2 (8.5 to 24.5)^a^	18.2 (7.2 to 30.2)^a^	−7.2 (−16.0 to 2.5)	−2.9 (−6.9 to 1.2)	−3.0 (−10.0 to 4.5)	−3.3 (−6.5 to 0.1)
Trend 2, years	2016 to 2023	2016 to 2023	2017 to 2023	2018 to 2023	2018 to 2021	NA
APC, % (95% CI)	4.9 (3.9 to 5.8)^a^	6.0 (4.6 to 7.4)^a^	8.3 (4.7 to 12.0)^a^	11.5 (8.2 to 14.8)^a^	5.7 (−16.5 to 33.8)	NA
Trend 3, years	NA	NA	NA	NA	2021 to 2023	NA
APC, % (95% CI)	NA	NA	NA	NA	−8.4 (−27.6 to 16.0)	NA
AAPC 2014-2023, % (95% CI)	7.3 (5.9 to 8.7)^a^	8.6 (6.6 to 10.6)^a^	2.9 (−0.2 to 6.0)	4.8 (2.9 to 6.8)^a^	−1.4 (−5.8 to 3.2)	−3.3 (−6.5 to 0.1)
AAPC comparison	−1.3 (−3.7 to 1.1)		−2.0 (−5.7 to 1.7)		1.8 (−3.5 to 7.1)	
Individuals with ALD, by sex	Female	Male	Female	Male	Female	Male
Trend 1, years	2014 to 2017	2014 to 2016	2014 to 2016	2014 to 2017	2014 to 2018	2014 to 2017
APC, % (95% CI)	12.7 (8.7 to 16.9)^a^	17.5 (6.3 to 29.8)^a^	−10.1 (−22.6 to 4.6)	−6.5 (−19.7 to 8.9)	−4.8 (−14.9 to 6.5)	−4.8 (−13.6 to 4.8)
Trend 2, years	2017 to 2023	2016 to 2023	2016 to 2023	2017 to 2023	2018 to 2021	2017 to 2021
APC, % (95% CI)	4.1 (2.8 to 5.4)^a^	5.3 (3.9 to 6.7)^a^	6.5 (4.4 to 8.7)^a^	9.8 (4.3 to 15.6)^a^	6.7 (−25.2 to 52.1)	2.5 (−6.9 to 12.8)
Trend 3, years	NA	NA	NA	NA	2021 to 2023	2021 to 2023
APC, % (95% CI)	NA	NA	NA	NA	−8.8 (−36.0 to 30.1)	−6.7 (−23.0 to 13.1)
AAPC 2014-2023, % (95% CI)	6.9 (5.7 to 8.1)^a^	7.9 (5.9 to 9.9)^a^	2.6 (−0.2 to 5.5)	4.1 (−0.7 to 9.1)	−2.1 (−8.5 to 1.4)	−2.1 (−5.1 to 1.0)
AAPC comparison	−1.0 (−3.3 to 1.3)		−1.5 (−7.1 to 4.2)		0.0 (−7.3 to 7.4)	

Abbreviations: AAPC, average annual percentage change; ALD, alcohol-associated liver disease; APC, annual percentage change; NA, not applicable.

^a^ Indicates statistical significance at *P* < .05.

## Discussion

This cohort study found increased population-level trends in postdischarge pharmacological cessation prescriptions and psychiatric referrals among hospitalized individuals with ALD, although the proportions remained low. Over the past decade, pharmacological cessation prescriptions have significantly increased, aligning with a Canadian study showing a 10-fold increase in AUD pharmacotherapy.^3^ In our study, psychiatric referrals and outpatient encounters encompassed general psychiatric evaluations, rather than being limited to alcohol-specific treatment referrals, due to limitations of dataset in identifying AUD-directed behavioral treatment. Despite this broad definition, psychiatric follow-ups remained low, accounting for only 6.5% in 2023. Individuals with cirrhosis had lower rates of pharmacotherapy cessation prescriptions and psychiatric referrals, likely due to concerns over impaired liver function and perceived limited medication options.^4^ Mortality rates peaked in 2020 and have since declined, possibly because of the COVID-19 pandemic and increased pharmacologic and psychiatric interventions since 2017. Whether mortality rates have reached an inflection point that heralds a sustained decline remains to be seen. Inherent limitations for database studies include potential misclassification bias from diagnosis coding and the inability to determine the specific indications for multiuse medications. As abstinence has been proven to reduce the risk of mortality and decompensation across all stages of ALD,^5^ our study provides crucial evidence of underutilization and trends of postdischarge pharmacological and psychiatric interventions, particularly among men and individuals with cirrhosis, highlighting the need for targeted strategies to support long-term alcohol cessation.

## Notes

Supplement 1.
eMethods
**eTable.** *ICD-9/10-CM* diagnosis codes and procedure codes
eReferences

Supplement 2.
Data Sharing Statement
