# Supplementary material for: Postdischarge Alcohol Cessation and Psychiatric Referrals in Alcoholic Liver Disease
Source: JAMA Netw Open. 2025 May 20;8(5):e2511619. doi: 10.1001/jamanetworkopen.2025.11619 (PMC12093184; doi:10.1001/jamanetworkopen.2025.11619)
Supplement: Supplement 1. — eMethods eTable. ICD-9/10-CM diagnosis codes and procedure codes eReferences [file jamanetwopen-e2511619-s001.pdf]

Supplemental Online Content

Chan SY, Yeo YH, Kim H, Polpichai N, Tsai YT, Ting PS. Postdischarge alcohol cessation and psychiatric referrals in alcoholic liver disease. *JAMA Netw Open*. 2025;8(5):e2511619. doi:10.1001/jamanetworkopen.2025.11619

eMethods
eTable. *ICD-9/10-CM* diagnosis codes and procedure codes
eReferences

This supplemental material has been provided by the authors to give readers additional information about their work.

© 2025 Chan SY et al. *JAMA Network Open*.

eMethods

Data source and ethical review

This population-based retrospective cohort study utilized de-identified electronic health records, encompassing diagnostic records, procedures, laboratory values data, medication prescriptions, and pharmacy genomic information within a real-time, real-world network (TriNetX, LLC, Cambridge, MA). The dataset spans from May 2003 to the present, which includes over 117 million individuals with and without insurance encompassing both inpatient and outpatient records from 65 healthcare organizations across the U.S. The database updates the patients' records every week. This network has been previously utilized and validated for retrospective population-level cohort studies [1]. The TriNetX platform complies with the Health Insurance Portability and Accountability Act (HIPAA) and is certified under the ISO 27001:2013 standard. All data were de-identified in accordance with Section §164.514(a) of the HIPAA Privacy Rule [2]. This study is exempt from informed consent and institutional review board approval as it is a retrospective secondary analysis of existing de-identified data and does not involve interaction/intervention with human subjects. This study adhered to the Strengthening the Reporting of Observational Studies in Epidemiology (STROBE) reporting guideline for cohort studies.

Study design, definitions and outcomes

Patients with alcohol-associated liver disease (ALD) were identified using the nineth or tenth revision Clinical Modification of International Classification of Diseases (ICD-9/10-CM) codes for alcoholic liver disease, alcoholic hepatitis (with and without ascites), and alcoholic cirrhosis (with and without ascites), alcoholic hepatic failure between January 1, 2014, and December 31, 2023 **(eTable 1)**. Patients under 21 years of age were excluded. Hospitalizations were defined using Current Procedural Terminology (CPT) codes for hospital inpatient care, observation, or inpatient consultation service within one month before or after ALD diagnosis. The index date was the first ALD diagnosis associated with hospitalization. The primary study outcomes were incidence of post-discharge (1) pharmacological cessation prescriptions including baclofen, naltrexone, acamprosate, disulfiram, gabapentin and topiramate, (2) psychiatric referrals or psychiatric outpatient encounters and (3) mortality rate on each year. Medications were identified through RxNorm codes, and psychiatric referrals or evaluations were determined using CPT and SNOMED codes. Annual incidence proportion (%) and incidence rate (cases/1000 person-years) of these outcomes were calculated. Incidence trends over 10 years were analyzed using joinpoint regression. We also estimated the mortality rate. Two subgroup analyses stratified patients by sex and cirrhosis status were performed. Non-cirrhotic ALD was defined as alcoholic liver disease or hepatitis without cirrhosis or decompensated manifestations (ascites, hepatic encephalopathy, or variceal bleeding).

Statistical analysis

© 2025 Chan SY et al. *JAMA Network Open*.

The annual incidence proportion and rate of primary outcomes were calculated using the TriNetX built-in platform. The denominator, representing hospitalized ALD patients, and the numerator, representing the primary outcomes, were determined separately for each year to ensure accurate annual calculations. Trends over the past 10 years were analyzed with piecewise log-linear regression models via Joinpoint Regression Software (version 5.2.0.0) [3]. Annual percentage change (APC) and average annual percentage change (AAPC), with 95% confidence intervals, were computed to identify significant shifts. Non-parallel AAPC comparisons were performed using permutation tests and parametric methods. To ensure robust analysis, a minimum of two observations was required from each joinpoint to the dataset's endpoints and between consecutive joinpoints, preventing any joinpoint from falling on a single observation [4].

eTable. ***ICD-9/10-CM*** **diagnosis codes and procedure codes**

**Disease**	***ICD-9/10-CM*** **diagnosis codes/procedure codes/ RxNorm codes**
ALD	K70, K70.1, K70.10, K70.11, K70.3, K70.30, K70.31, K 70.4, K70.9,
Hospitalization	1013659, 1013682, 1013660, 99251, 99253, 99254, H009, H008, Visit: inpatient encounter
Psychiatric referral or outpatients encounter	1012682, 1031023, 99492, 99493, 90791, 90792, 90887, 94.13, 308477009, Z04.6
Pharmacology cessation prescriptions	1292, 3554, 7243, 82819, 25480, 38404
ALD with cirrhosis	K70.3, K70.30, K70.31
ALD without cirrhosis	K70, K70.1, K70.10, K 70.4, K70.9 (excluding K70.3, R18, K76.82, I85)

eReferences

1. Liu, B.D., et al., *Shorter-acting glucagon-like peptide-1 receptor agonists are associated with increased development of gastro-oesophageal reflux disease and its complications in patients with type 2 diabetes mellitus: a population-level retrospective matched cohort study*. Gut, 2024. **73**(2): p. 246-254.
2. Code of Federal Regulations. *§ 164.514 Other requirements relating to uses and disclosures of protected health information*. 2024 [cited 10th December 2024; Available from: https://www.ecfr.gov/current/title-45/subtitle-A/subchapter-C/part-164/subpart-E/section-164.514.
3. National Cancer Institute. *Joinpoint Trend Analysis Software. Surveillance Research Program US National Cancer Institute*. 2024 [cited 3rd November 2024]; Available from: https://surveillance.cancer.gov/joinpoint/.

© 2025 Chan SY et al. *JAMA Network Open*.

4. Meza, R., E. Jimenez-Mendoza, and D.T. Levy, *Trends in Tobacco Use Among Adolescents by Grade, Sex, and Race, 1991-2019.* JAMA Netw Open, 2020. **3**(12): p. e2027465.

© 2025 Chan SY et al. *JAMA Network Open*.
