# Supplementary material for: Postdischarge Alcohol Cessation and Psychiatric Referrals in Alcoholic Liver Disease
Source: JAMA Netw Open. 2025 May 20;8(5):e2511619. doi: 10.1001/jamanetworkopen.2025.11619 (PMC12093184; doi:10.1001/jamanetworkopen.2025.11619)
Supplement: Supplement 2. — Data Sharing Statement [file jamanetwopen-e2511619-s002.pdf]

Data Sharing Statement

Chan. Postdischarge Alcohol Cessation and Psychiatric Referrals in Alcoholic Liver Disease. *JAMA Netw Open*. Published May 20, 2025. doi:10.1001/jamanetworkopen.2025.11619

Data

Data available: No

Additional Information

Explanation for why data not available: TriNetX is a subscription based platform, not publically available. It would not be our place to share this data. Interested parties are able to use the platform for the posted fees by TriNetX.
